# The long-lasting post-stimulation inhibitory effects of bladder activity induced by posterior tibial nerve stimulation in unanesthetized rats

**DOI:** 10.1038/s41598-020-76987-2

**Published:** 2020-11-16

**Authors:** Eunkyoung Park, Jae-Woong Lee, Taekyung Kim, Minhee Kang, Baek Hwan Cho, Jiho Lee, Sung-Min Park, Kyu-Sung Lee

**Affiliations:** 1 Biomedical Engineering Research Center, Samsung Medical Center, Sungkyunkwan University School of Medicine, Seoul, Korea; 2 Department of Medical Device Management and Research, SAIHST, Sungkyunkwan University, Seoul, Korea; 3 Department of Urology, Samsung Medical Center, Sungkyunkwan University School of Medicine, Seoul, Korea; 4 Department of Creative IT Engineering, Pohang University of Science and Technology (POSTECH), Pohang, Korea

**Keywords:** Bladder, Biomedical engineering, Urinary incontinence, Preclinical research, Urological manifestations

## Abstract

Tibial nerve stimulation (TNS) is one of the neuromodulation methods used to treat an overactive bladder (OAB). However, the treatment mechanism is not accurately understood owing to significant differences in the results obtained from animal and clinical studies. Thus, this study was aimed to confirm the response of bladder activity to the different stimulation frequencies and to observe the duration of prolonged post-stimulation inhibitory effects following TNS. This study used unanesthetized rats to provide a closer approximation of the clinical setting and evaluated the changes in bladder activity in response to 30 min of TNS at different frequencies. Moreover, we observed the long-term changes of post-stimulation inhibitory effects. Our results showed that bladder response was immediately inhibited after 30 min of 10 Hz TNS, whereas it was excited at 50 Hz TNS. We also used the implantable stimulator to observe a change in duration of the prolonged post-stimulation inhibitory effects of the TNS and found large discrepancies in the time that the inhibitory effect lasted after stimulation between individual animals. This study provides important evidence that can be used to understand the neurophysiological mechanisms underlying the bladder inhibitory response induced by TNS as well as the long-lasting prolonged post-stimulation effect.

## Introduction

Idiopathic overactive bladder (OAB) is described as a urinary urgency, usually accompanied by increased daytime frequency and/or nocturia, with urinary incontinence (OAB‐wet) or without (OAB‐dry), in the absence of urinary tract infection or other obvious pathology^1–3^. It is not typically associated with the presence of a urinary tract infection or urgency incontinence. In the United States, OAB is prevalent in about 17% of people older than 40 years, whereas 30–40% of people over the age of 75 suffer from this disorder^4,5^. Pharmacotherapy for OAB involves the use of antimuscarinic drugs or beta-3 agonists to inhibit bladder contraction. However, these frequently cause side effects, such as mouth dryness, constipation, blurred vision, nausea, and gastrointestinal and cognitive dysfunctions. As a result, 20–50% of patients are not satisfied with the treatment^6,7^.

Neuromodulation therapy is an effective alternative solution involving electrical stimulation of specific nerves to modulate dysfunctional bladder activity. In recent clinical applications, neuromodulation treatment showed about the same efficacy as pharmaceutical treatment, but with less severe adverse effects^8^. One method of neuromodulation is sacral nerve stimulation, which can relieve lower urinary tract symptoms such as frequent urination, urge incontinence, and urinary retention^9,10^. Sacral nerve stimulation modulates the micturition reflex by electrically stimulating the afferent sacral nerve, interfering with sensory input from the bladder to the pontine micturition center. A pulse generator, surgically implanted in the sacral spinal region, provides continuous stimulation^11^.

Tibial nerve stimulation (TNS) has been suggested as an alternative approach to the modulation of bladder function. It involves placing a fine needle electrode near the posterior tibial nerve through the skin on the ankle. Electrical stimulation of the afferent fibers of tibial nerves is delivered to the L4–S3 nerve roots. These are innervated to the urinary sphincter and bladder, and hence control urinary function by inhibiting the sensory input from the bladder^12–14^. The standard protocol is 30 min every week for 3 months in a clinical setting. This is then followed by another series of stimulations to maintain the effect^8,15^. Current TNS has demonstrated overall treatment efficacy in the clinical setting. In a small-scale, double-blind, randomized controlled trial, 71% of patients who received PTNS (n = 18) and 0% of patients who received placebo (n = 17) demonstrated clinical efficacy^16^. In a multicenter clinical trial including 220 men and women with OAB receiving 12 weeks of therapy, the PTNS group showed greater significant improvement in overall bladder symptoms as compared with the sham group (54.5% vs. 20.9%, respectively). A recent retrospective study of 183 patients with idiopathic OAB over 9 years reported that 25.4% of patients (43 of 169) achieved ≥ 75% symptom relief and 61.5% of patients (104 of 169) achieved ≥ 50% symptom relief^17^. However, although current TNS has overall clinical efficacy, a large discrepancy (37%–86%) in its treatment success has been reported^18–21^.

TNS has a prolonged post-stimulation inhibitory effect, although the effects of sacral nerve stimulation diminish immediately after stimulation ends. However, the underlying mechanism of the post-stimulation inhibitory effect of TNS is not fully understood. Only a few animal studies and clinical trials have investigated the prolonged post-stimulation inhibitory effect of TNS. Tai et al. reported that 3 to 5 min of TNS at both 5 Hz and 30 Hz effectively inhibited reflex bladder activity in anesthetized cats^22^. The inhibitory effect was found to persist after stimulation at these frequencies. The authors also showed that continuous stimulation for 30 min induced both a prolonged inhibition of bladder activity after stimulation and a significantly increased bladder capacity that lasted 2 h. A similar post-stimulation inhibitory effect was shown in anesthetized rats^23^. Here, 30 min of 5 Hz TNS led to post-stimulation inhibition and increased bladder capacity that lasted for 50 min after stimulation. By contrast, clinical reports demonstrated that TNS has a prolonged post-stimulation inhibitory effect that lasts a few days and even weeks^13,24,25^. The discrepancy in the results of animal and clinical studies is due to the difficulties inherent in replicating a clinical setting in animal studies. In particular, anesthesia is required in animal studies to monitor bladder activity, whereas, in clinical studies this can be monitored over prolonged periods without the use of anesthesia. It is difficult to obtain reliable data of the prolonged TNS effects in longer duration experiments using anesthetized animals as the micturition reflex is very sensitive to anesthesia^26–28^.

In this study, a miniature, fully implantable stimulator was developed to administer TNS in small animals without the use of anesthetics. The purpose of this study is to use this stimulator to confirm the response of bladder activity to the different stimulation frequencies and also to observe the duration of prolonged post-stimulation inhibitory effects after TNS.

## Results

### Frequency dependence of TNS effects

**Figure 1:**
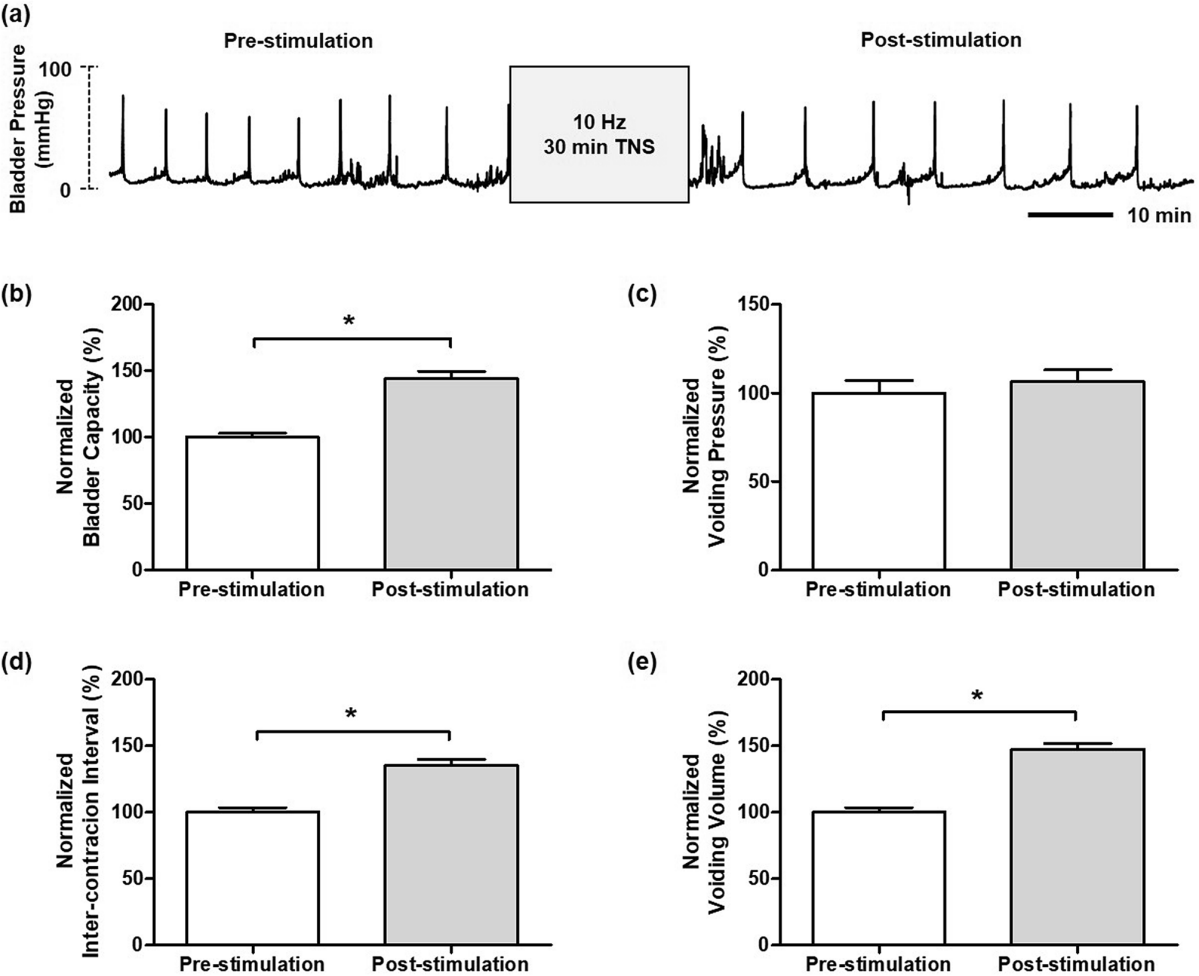
The pre- and post-stimulation effects of the 30 min 10 Hz TNS. (**a**) Representative CMG traces of pre- and post-stimulation of 30 min at 10 Hz. (**b**) bladder capacity, (**c**) voiding pressure, (**d**) inter-contraction interval, (**e**) voiding volume. The normalized data are presented as a mean with the standard error. The significance of the differences between the pre- and post-stimulation results were determined by the Student’s t-test (*P < 0.05).

**Figure 2:**
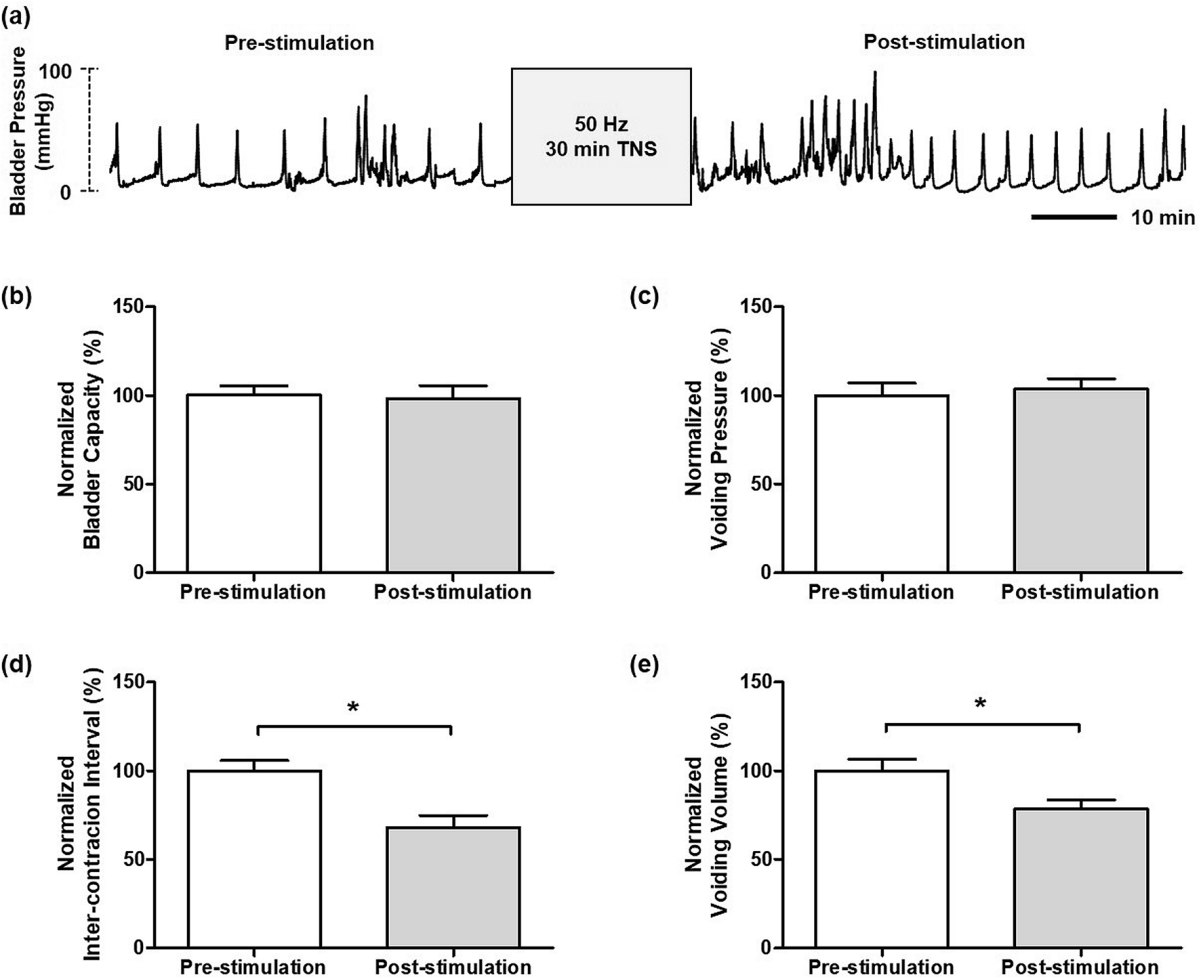
The pre- and post-stimulation effects of the 30 min 50 Hz TNS. (**a**) Representative CMG traces of pre- and post-stimulation of 30 min at 10 Hz. (**b**) bladder capacity, (**c**) voiding pressure, (**d**) inter-contraction interval, (**e**) voiding volume. The normalized data are presented as a mean with the standard error. The significance of the differences between the pre- and post-stimulation results were determined by the Student’s t-test (*P < 0.05).

When CMGs were stable, 30 min of stimulation at 10 Hz on the tibial nerve significantly inhibited bladder activity in the unanesthetized rats. As shown in Fig. 1, stimulation at 10 Hz frequency (n = 6) resulted in a significant increase (*P* < 0.05) in BC and VV to 144.1 ± 5.07% and 147.5 ± 4.24% of pre-stimulation respectively, as well as a significantly longer duration of effects (*P* < 0.05) on ICI to 135.3 ± 4.60% of pre-stimulation. However, no significant difference in VP (106.6 ± 4.64%; *P* = 0.236) before and after stimulation was found. By contrast, TNS at 50 Hz (n = 6) resulted in a significant excitation in bladder activity after 30 min of stimulation. Though BC (98.4 ± 7.23%; *P* = 0.815) and VP (103.7 ± 5.88%; *P* = 0.107) at 50 Hz showed no change compared to the pre-stimulation results, significant decreases in both ICI and VV (68.1 ± 6.68% and 78.5 ± 5.05%, respectively; *P* < 0.05) were observed as shown in Fig. 2.

### Prolonged post-stimulation effects

**Figure 3:**
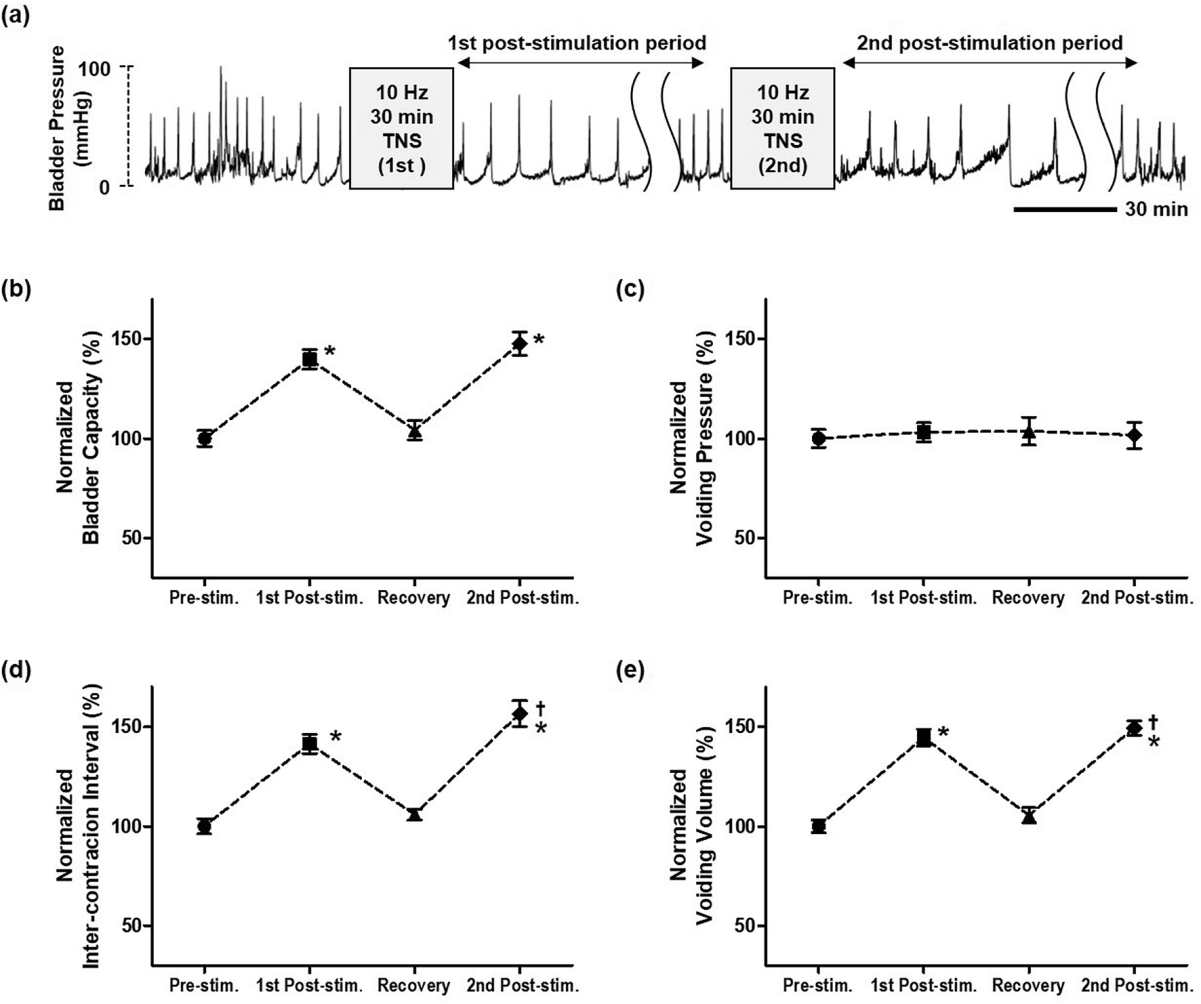
The prolonged post-stimulation effects of the 30 min 10 Hz TNS. (**a**) Representative CMG traces of post-stimulation effects. By monitoring the CMG, it was confirmed that the average duration of the post-stimulation effects was 10.2 ± 1.06 h (range: 3.2 h to 24.6 h, n = 12). When the first prolonged post-stimulation effects diminished, a second TNS was performed using the same stimulation parameters. After the second TNS, the prolonged post-stimulation period lasted for 8.6 ± 1.24 h (range: 2.8 h to 22.5 h, n = 7). The (**b**) bladder capacity, (**c**) voiding pressure, (**d**) inter-contraction interval, and (**e**) voiding volume were monitored for the prolonged post-stimulation effect periods. The normalized data are presented as a mean with standard error. Significant differences of all data were demonstrated using ANOVA with Bonferroni multiple comparison. *P < 0.05 was considered statistically significant compared with the pre-stimulation period, and ^†^*P* < 0.05 was considered statistically significant compared with the first post-stimulation period.

CMGs of the 12 unanesthetized rats were continuously monitored after 30 min of 10 Hz TNS. As shown in Fig. 3, 10 Hz TNS induced inhibitory effects of bladder contraction as well as long-lasting post-stimulation effects. The duration of prolonged post-stimulation effects was measured as the time taken for both the ICIs and VVs to return to the levels observed during the pre-stimulation period. As a result, the prolonged post-stimulation inhibitory effects lasted for 10.2 ± 1.06 h (range 3.2 h to 24.6 h, n = 12). The BC, ICI, and VV (139.8 ± 4.97%, 141.4 ± 4.75%, and 144.6 ± 4.17% respectively; *P* < 0.05) were significantly increased in comparison to the pre-stimulation period (Fig. 3b–d). However, the VP (103.2 ± 4.79%; *P* = 0.480) was not significantly changed during the prolonged post-stimulation effects (Fig. 3e).

When the first prolonged post-stimulation effects diminished, a second TNS was immediately administered. The second prolonged post-stimulation period lasted for about 8.6 ± 1.24 h (range: 2.8 h to 22.5 h, n = 7) and did not significantly differ from the first prolonged post-stimulation period. Additionally, the BC, ICI, and VV (147.7 ± 5.96%, 156.6 ± 6.52% and 149.4 ± 3.61%; respectively; *P* < 0.05) were significantly increased, in a similar fashion to that observed for the first prolonged post-stimulation effects (Fig. 3b–d). The ICI (*P* = 0.002) and VV (*P* = 0.019) showed even greater increases in second post-stimulation period than those of the first post-stimulation period (Fig. 3c,d). By contrast, the VP did not change significantly (*P* = 0.450) in CMGs measured over the prolonged period after the second TNS (Fig. 3e).

## Discussion

This study used unanesthetized rats to provide a closer approximation of the clinical setting and evaluated the changes in bladder activity in response to 30 min of TNS with low and high frequencies. Additionally, a fully implantable stimulator was used to observe the long-term changes of post-stimulation inhibitory effects while the rats were unanesthetized.

Previous animal studies showed that TNS at low frequencies can induce an inhibitory effect. Feline and rodent studies also demonstrated inhibition effects with 5, 10, 20, and 30 Hz TNS^22,23,29–31^. In particular, a recent study using rats reported that TNS at 10 Hz produced a maximal inhibitory effect and inhibited bladder contractions immediately after the start of stimulation^30^. Sacral nerve stimulation at low frequency (10–14 Hz) has also been shown to have a strong inhibitory effect on bladder contraction in clinical applications^32,33^. Our findings were consistent with those of previous studies, confirming that 10 Hz TNS had an inhibitory effect on bladder contraction without a change in voiding pressure. Choudhary and colleagues reported that TNS significantly increases bladder compliance and volume without any changes in pressure during voiding contraction in anesthetized rats with bladder overactivity induced by acetic acid^31^. Moreover, they suggested that TNS increases bladder storage capacity by delaying the onset of voiding by inhibiting bladder afferent signaling at the peripheral site. Thus, it is possible that low frequency TNS exerts an inhibitory effect on afferent signaling by eliciting reflex activity in the sympathetic inhibitory pathways to suppress OAB. Our study found an excitatory bladder reflex after TNS at 50 Hz as borne out by previous studies which showed that higher frequencies evoked the excitatory reflex in comparison to the typical values that inhibit bladder activity (5–30 Hz)^34–36^. Even in pudendal nerve stimulation studies, there have been reports that low frequency stimulation produces inhibition, and high-frequency stimulation produces excitation of bladder activity^37–40^. Therefore, the role of the pudendal afferent nerve can be hypothesized to be important in inducing an excitatory reflex by 50-Hz TNS. Anatomically, the posterior tibial nerve is a branch of the sciatic nerve that carries spinal roots L4-L6 and S1-S3 as the largest distal extension of the lumbosacral plexus. The pudendal nerve also originates from the sacral plexus along with the sciatic nerve^41^. Thus, our hypothesis can be supported by the role of the pudendal nerve, as it can interact with the stimulation signal from the tibial nerve. Our hypothesis is also supported by the fact that the voiding efficiency in the anesthetized rats was decreased by cutting the pudendal nerve and that the bladder excitatory reflexes in the patient with spinal cord injury were induced by pudendal afferent nerve stimulation^39,42^.

**Figure 4:**
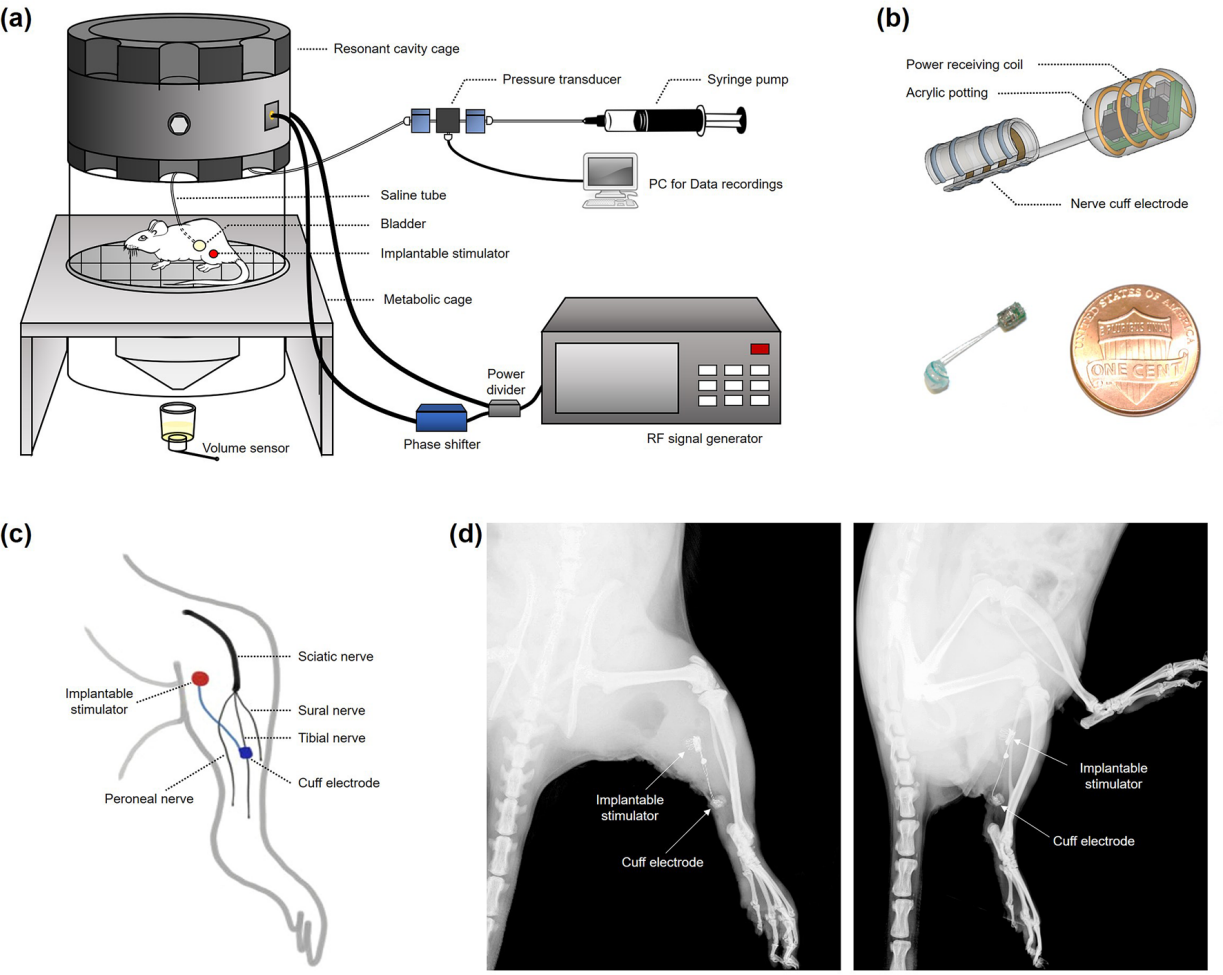
(**a**) The experimental system. TNS was carried out on unanesthetized rats utilizing an implantable electrical stimulator and wireless power delivery system. TNS and CMG were simultaneously performed in a metabolic cage. (**b**) Size comparison of the implantable electrical stimulator. The implantable electrical stimulator was designed as a small-scale implant (3.7 mm l × 2.3 mm w; max. height of coil: 3.0 mm) to which a 2 mm custom-fabricated platinum nerve cuff electrode was attached. (**c**) Device placement. The implantable electrical stimulator was placed near the gastrocnemius muscle, and the nerve cuff electrode was placed precisely on the tibial nerve as one of the three branches of the Sciatic nerve. (**d**) X-ray images used to identify the implantable stimulator.

In this study, we used a fully implantable TNS system, modified and refined from the system developed by Montgomery et al., to observe the prolonged effects after 30 min of TNS without anesthesia, as shown in Fig. 4^43^. We observed that the prolonged post-stimulation effects were maintained for 10.2 ± 1.06 h (range: 3.2 h to 24.6 h, n = 12) after 10 Hz TNS for 30 min (Fig. 3a). Additionally, when a second TNS was performed, after the effects of the first prolonged post-stimulation had diminished, a prolonged post-stimulation period (8.6 ± 1.24 h; range: 2.8 h to 22.5 h; n = 7) of similar duration to the first prolonged post-stimulation period was observed, as shown in Fig. 3a. Unlike sacral nerve stimulation or pudendal nerve stimulation, TNS offers a prolonged duration of post-stimulation effects. This implies that TNS has a different mechanism of action to sacral nerve or pudendal nerve stimulation. Hence a better understanding of the therapeutic aspects of TNS with regard to bladder contraction is required. Typically, the persistent inhibitory effects of bladder activity with TNS are dependent on the central neural pathways. The neural circuit responsible for controlling micturition is located on the pontine micturition center^44–46^. Thus, the increased bladder capacity induced by 10 Hz TNS, as shown in Figs. 1b and 3b, implies that either the gating circuit of this center is directly modulated, or the afferent input to that circuit is suppressed. This may also explain the increase in VV and ICI corresponding to the increase in BC, as shown in our results for 10 Hz TNS. In recent studies, the roles of various inhibitory neurotransmitter receptors for TNS bladder inhibition have also been reported. In particular, the activation of opioid receptors has been suggested to have an essential role in the inhibition of bladder activity by TNS. In previous researches, the naloxone, an opioid receptor antagonist, was reported to block the inhibitory effects of bladder overactivity^47,48^. This demonstrates that opioid receptors play an important role in the treatment of bladder overactivity. In addition, in studies with three subtypes (μ, κ, and δ) of opioid receptors in TNS inhibition, subtype μ and κ receptors were reported to play major roles in the modulation of bladder activity^49^. These results suggested that it can be postulated that the activation of opioid receptors is responsible for bladder overactivity treatment by TNS. Gamma-aminobutyric acid (GABA) is also a major neurotransmitter that induces inhibitory effects via spinal and supraspinal micturition reflexes^50,51^. Previous studies revealed an association between spinal GABA receptor activation and its effects on bladder overactivity. Moreover, these studies reported that GABA agonists attenuated bladder overactivity and suggested that this mechanism could be effective in treating bladder disorders caused by C-fiber afferent activation^52–54^. Thus, understanding of mechanisms for bladder activity that are explained by neurotransmitters might provide opportunities to develop novel OAB treatment methods by TNS.

Previous animal studies using rats and cats have reported that prolonged post-stimulation inhibition of bladder activity can be induced by TNS, with the effects maintained for 50 min to 2 h after stimulation^22,23,34^. However, clinical studies show that the prolonged post-stimulation inhibitory effects of TNS persist for significantly longer (a few days or weeks) than animal studies^13,15^. This noticeable difference may be attributed to shorter experimental times and the use of anesthetized animals that were not similar to the clinical setup. As a result, direct comparison of the results between animal and clinical studies presents challenges. In particular, the anesthetics used in prolonged animal studies are known to sensitively influence the micturition reflex, and alter synaptic transmission. For example, urethane, an anesthetic commonly used in long-term animal experiments, is known to affect glutamatergic synaptic transmission in the brain stem and spinal cord^55,56^. Therefore, urethral activity for voiding and bladder contraction pressure will be sensitive to anesthetics such as urethane.

Some previous studies on the effects of TNS used OAB animal models induced that were chemically induced via irritation with 0.2%–0.5% acetic acid^31,57^. Choudhary et al. showed the effect before and after TNS using the normal rat model^57^. Thereafter, bladder overactivity was induced with acetic acid in the same rats and the effects before and after TNS were investigated. They reported that the normal model did not exhibit changes in the urodynamics before and after TNS; however, the OAB model exhibited increased bladder capacity and filling time without changes in voiding pressure after TNS. On the contrary, other studies using a normal model showed increased bladder compliance and contraction duration without changes in voiding pressure after TNS, similar to that in studies with an OAB model^23,30,34,58^. Although the OAB model induced with acetic acid exhibited increased urinary urgency, frequency, and nocturia, these symptoms were similar to those of cystitis. Since cystitis and OAB are known to have different mechanisms, studies on the effect of TNS using the OAB model induced by acetic acid may have limitations in terms of explaining the effect of TNS in patients with OAB in clinical practice.

Interestingly, we found a large discrepancy in the time that inhibitory effects lasted after stimulation for each animal. These results suggest that a personally optimized stimulation protocol, taking the varying responses of patients into account, is needed, rather than a standard treatment protocol of a 30 min stimulation per week. Recent studies have also demonstrated that varying the stimulation method clinically improved the results compared to the standard protocol^59–61^. Finazzi et al. demonstrated that more frequent stimulation sessions can achieve clinical therapeutic effects faster^62^. However, current TNS systems have the disadvantage of requiring frequent visits to hospitals for TNS treatment, and the risk of pain and infection due to repetitive needle electrode insertion. In order to overcome these drawbacks, research on implantable stimulators has been actively pursued. Heesakkers et al. used an implantable tibial nerve stimulator and a novel stimulation protocol consisting of six sessions per week for the first 3 months, followed by three sessions per week for the following 3 months^63^. They reported clinical success in patients with OAB, urge incontinence, and urgency frequency after 6 months in the first clinical application. Another study with different implantable stimulator reported its clinical effects on OAB urgency and urinary incontinence observed from 3-month and 6-month follow-ups^64^. Recently, in their 3-year follow-up study, Dorsthorst et al. confirmed both the safety (without any technical failures) and clinical efficacy of this treatment for the improvement of patient quality of life^65^. Therefore, if an implantable stimulator is helpful in treatment of OAB patients, new stimulation protocols for an implantable stimulator need to be developed by taking into consideration the duration of the prolonged post-stimulation inhibitory effect in individual patients.

In conclusion, this study provides important evidence that can be used to understand the neurophysiological mechanisms of the prolonged post-stimulation inhibitory effect induced by TNS. It further indicates the need to accurately mimic the clinical setting in animal studies, in order to correlate the results to clinical application. Furthermore, both urodynamic and electrophysiological studies should be conducted to facilitate a better understanding of the TNS mechanism. We also suggest that analysis of individual patients' responses to stimuli is necessary to obtain better therapeutic effects in OAB treatment with TNS. Further research on implantable stimulators based on closed-loop control is needed to provide optimal stimulation treatment for individual patients. This may provide an opportunity to overcome the current limitations of OAB treatment using TNS.

## Materials and methods

The experimental and surgical procedures performed on the animals were reviewed and approved by the Institutional Animal Care and Use Committee of the Samsung Medical Center, Korea (No. 20190925001). The study was carried out in accordance with the National Institutes of Health Guide for the Care and Use of Laboratory Animals.

### Fully implantable stimulator

To observe the prolonged inhibitory effects of bladder activity after TNS in unanesthetized rats, a miniature wireless stimulator was implanted into each freely moving small animal. Recently, several research groups have developed small, wireless, and fully implantable devices to deliver optical stimulation in optogenetic studies^43,66^. In particular, Montgomery et al. developed a novel optical stimulation device, which was small in size (10 mm^3^) and lightweight (20 mg)^43^. This optical stimulation system can be wirelessly powered by radio frequency within a resonant cavity cage. The miniature optoelectronic device consists of an implantable circuit for receiving power, a micro-LED for optical stimulation, and an external circuit for wireless power transmission. Importantly, this device allowed optical stimulation of the central or peripheral nerve in freely moving small animals. In this study, we developed a fully implantable stimulator based on the miniature device developed by Montgomery et al. albeit further modified and refined as shown in Fig. 4a. The power transmission component of the wireless TNS system consists of a radio frequency (RF) signal generator, an RF power amplifier, a power divider, a phase shifter, and an RF resonant cavity cage. Since the RF energy is contained in the RF resonant cavity cage, it can have a high Q factor and wirelessly deliver power to the implanted stimulator. Implantable stimulators include a printed circuit board (PCB), coils, and a cuff electrode (Fig. 4b). The coil consists of 0.09 mm diameter wires which receive wireless power. The PCB consists of a simple circuit made up only of capacitors and diodes (3.7 mm l × 2.3 mm w; max. height with coil: 3.0 mm). We also used a custom-made nerve cuff electrode (MicroProbes, Gaithersburg, MD, USA) consisting of two thin stainless-steel wires to electrically stimulate the tibial nerve in a fully implanted setting. This device allows us to locally and accurately stimulate only the tibial nerve, and overcome the issues surrounding the use of conventional needle electrodes that often stimulate other nerves around the tibial nerve.

### Experimental setup

The experiment was conducted with 24 female Sprague–Dawley rats (weights ranging 280–310 g). The rats were anesthetized by inhaling isoflurane (concentrations ranging 3–5%, O_2_ level: 0.1 L/min). The rats were kept under anesthesia during the implantation of the stimulator and the bladder catheterization procedure. The posterior tibial nerve was exposed on the medial side of the left hind limb above the ankle. The nerve cuff electrode was mounted onto the tibial nerve and the skin was sutured over both the cuff and the stimulator (Fig. 4c,d). The bladder was then exposed by a midline incision on the abdomen, and a catheter inserted via the bladder dome. The opposite end of the catheter was closed off to prevent urinary leakage and fixed under the skin to prevent any possible damage by the rats. Four days later, the catheter was connected to a pump for saline infusion and to a pressure transducer to record bladder contraction and pressure.

### Stimulation protocol and data acquisition

The inserted nerve cuff electrode delivered rectangular pulses with a 200 μs pulse width to the tibial nerve. The stimulation time was set to 30 min to mimic a typical clinical application^8,13,15^. The threshold (T) of stimulation intensity was set by observing toe twitches. In previous studies of both cats and rats, TNS with an intensity greater than 2 T was sufficient for inhibiting reflex bladder contractions^23,30^. Therefore, we applied TNS intensity between 2 and 3 T to inhibit the bladder. A cystometrogram (CMG) was performed on the unanesthetized rats by the continuous infusion of body-warm saline at a rate of 0.1 ml/min until inter-contraction intervals stabilized. To determine whether TNS influenced urinary function, we measured the pre- and post-stimulation: bladder capacity (BC), voiding pressure (VP), inter-contraction interval (ICI), and voiding volume (VV). CMGs were continuously performed during all experimental procedures using LabChart software and a PowerLab data acquisition system (AD Instruments, Colorado Springs, CO, USA).

In the first experiments, the tibial nerve was stimulated at either 10 Hz (n = 6) or 50 Hz (n = 6) to determine the most effective frequency for inhibiting bladder activity. CMGs were performed before, and after 30 min of TNS at different frequencies and included at least five bladder contractions to determine which frequency induced the greatest effect on bladder contractions. A second round of experiments was performed to confirm the changes in post-stimulation effect. After the 30 min TNS, using the stimulation frequency determined in the first experiment, the prolonged post-stimulation effects were monitored in 12 unanesthetized rats. Post-stimulation inhibitory effects were considered to have disappeared when both ICI and VV, measured in real time, were equal to the values measured in the pre-stimulation session. After the post-stimulation effects disappeared, a second round of 30 min TNS were administered to randomly selected rats (n = 7) and the post-stimulation effects observed.

### Data analysis

All data were analyzed using LabChart software and MATLAB (MathWorks, Torrance, CA, USA) was used for additional analysis. The measured data from CMGs were normalized by the parameters obtained in the pre-stimulation period (before TNS) and the change expressed as a percentage of the value for that parameter established in the pre-stimulation period (the control). All the data are presented as mean and standard error. Statistical analyses were performed using either the Student t-test or ANOVA followed by Bonferroni multiple comparison, where a value of *P* < 0.05 was considered significant.
